# Supplementary material for: Content-rich biological network constructed by mining PubMed abstracts
Source: BMC Bioinformatics. 2004 Oct 8;5:147. doi: 10.1186/1471-2105-5-147 (PMC528731; doi:10.1186/1471-2105-5-147)
Supplement: Additional File 2 — The original results of the above study (non-essential files are deleted to keep the file size under the limit set by BMC bioinformatics). [file 1471-2105-5-147-S2.bz2 › chilibotAdditionalFile2/dip50/230ID7931156E749/html/UL5_UL8.html]

 


 **UL5** and **UL8** 
  
Found 42 abstracts in PubMed, retrieved 42.  
 

 What does Google say? 
 PDF only 
| .edu only 

---

**Interactive relationship** (e.g. stimulation, inhibition, etc)

**Neutral relationship**- The interactions of the UL52 protein with the  **UL8**  and  **UL5**  proteins were analysed by using the yeast two hybrid system.  Ref: 10501495 J Gen Virol, 1999
- Inhibition of herpes simplex virus replication by a 2 amino thiazole via interactions with the helicase component of the  **UL5**   **UL8**  UL52 complex.  Ref: 9696789 J Virol, 1998
- Surface plasmon resonance measurements demonstrated an interaction between ICP8 and the  **UL5**  52 8 heterotrimer but not with the  **UL5**  52 subassembly or the  **UL8**  protein alone.  Ref: 9278436 J Biol Chem, 1997
- Experiments performed using extracts from doubly infected cells indicated that  **UL8**  could interact separately with both the  **UL5**  and UL52 proteins.  Ref: 7931156 J Gen Virol, 1994
- These data suggest that  **UL8**  protein mediates an interaction between the  **UL5**  52 core enzyme and ICP8 that optimizes the utilization of ICP8 covered DNA templates during DNA replication.  Ref: 8702953 J Biol Chem, 1996
- Reconstitution of lagging strand synthesis in the presence of  **UL5**  UL52 was achieved by the addition of partially purified  **UL8** .  Ref: 1321275 J Virol, 1992
- On the other hand, the mutant protein retains its ability to interact with  **UL5**  as indicated by copurification and with  **UL8**  as indicated by a supershifted band in the gel mobility shift assay.  Ref: 10075707 J Biol Chem, 1999
- We demonstrate here that the  **UL8**  protein is required for the helicase activity of the  **UL5**  52 subassembly on long duplex DNA substrates >30 nucleotides with a single stranded DNA loading site fully coated with the virus encoded single strand DNA binding protein, ICP8.  Ref: 9278436 J Biol Chem, 1997
- To determine the role of  **UL8**  in primase assays, the activity of the  **UL5**  52 subassembly was compared to that of the heterotrimer reconstituted by adding  **UL8**  to  **UL5**  52.  Ref: 8106478 J Biol Chem, 1994
- Deletion mutants of the herpes simplex virus type 1  **UL8**  protein effect on DNA synthesis and ability to interact with and influence the intracellular localization of the  **UL5**  and UL52 proteins.  Ref: 9344911 Virology, 1997
- MAb 0811 was shown to recognize the  **UL8**  protein in both Western blots and immunoprecipitation assays and to co precipitate the other two proteins in the complex from insect cells triply infected with recombinants expressing the  **UL5** ,  **UL8**  and UL52 polypeptides.  Ref: 7931156 J Gen Virol, 1994
- Moreover,  **UL8**  protein was required to reverse the inhibitory effect of ICP8 on the DNA dependent ATPase and primase activities of the  **UL5**  52 proteins.  Ref: 8702953 J Biol Chem, 1996
- These results suggest that a function of the  **UL8**  component of the HSV helicase primase complex is to increase the efficiency of primer synthesis by  **UL5**  52.  Ref: 8106478 J Biol Chem, 1994
- The  **UL8**  component of the herpes simplex virus helicase primase complex stimulates primer synthesis by a subassembly of the  **UL5**  and UL52 components.  Ref: 8106478 J Biol Chem, 1994
- We found that while ICP8 stimulated the DNA helicase activity of the  **UL5**  52 proteins up to 3 fold, maximum stimulation by ICP8 required the presence of  **UL8**  protein.  Ref: 8702953 J Biol Chem, 1996

**Non-interactive relationship** (e.g. studied together, co-existance, homology, etc.)

- The DNA helicase primase complex of HSV 1 consists of three sub units with molecular weights of 1 0 00 and 7 00, encoded by genes UL52,  **UL5**  and  **UL8** , respectively.  Ref: 9421876 Virus Genes, 1997
- Herpes simplex virus type 1 encodes a helicase primase complex composed of the products of the  **UL5** , UL52, and  **UL8**  genes.  Ref: 8662872 J Biol Chem, 1996
- Since this pattern can form in cells infected with mutants which are defective in  **UL5** ,  **UL8** , UL9, or UL52 in the presence of polymerase inhibitors C.  Ref: 9151871 J Virol, 1997
- Although the  **UL5**  52 subassembly functions in coupled primase polymerase assays on homopolymeric templates, its activity on natural DNA templates has been reported to require  **UL8** .  Ref: 8106478 J Biol Chem, 1994
- To improve our ability to study these proteins, we have expressed  **UL5** ,  **UL8** , UL9, and UL52 in insect cells by using the baculovirus expression system.  Ref: 2535726 J Virol, 1989
- All three subunits of this complex  **UL5** ,  **UL8** , and UL52 are required for viral DNA replication in all cell types.  Ref: 9445019 J Virol, 1998
- Herpes simplex virus type 1 HSV 1 encodes a helicase primase that consists of three polypeptides encoded by the  **UL5** ,  **UL8** , and UL52 genes Crute, J.J.  Ref: 2556383 J Biol Chem, 1989
- These studies reveal that in the absence of ICP27, expression of the  **UL5** ,  **UL8** , UL52, UL9, UL42, and UL30 genes is significantly reduced at the level of mRNA accumulation.  Ref: 8627723 J Virol, 1996
- The herpes simplex virus type 1 HSV  **UL5** ,  **UL8** , and UL52 proteins form a helicase primase complex in infected cells.  Ref: 8106478 J Biol Chem, 1994
- T157602 inhibited reversibly the helicase activity of the HSV  **UL5**   **UL8**  UL52  **UL5**  8 52 helicase primase complex with an IC50 concentration of compound that yields 50% inhibition of 5 microM.  Ref: 9696789 J Virol, 1998
- We constructed recombinant viruses based on the herpes simplex virus type 1 mutant tsK which individually were able to express the products of four viral DNA replication genes  **UL5** ,  **UL8** , UL9 and UL52 in the absence of any of the other proteins required for viral DNA synthesis.  Ref: 1312116 J Gen Virol, 1992
- However the addition of  **UL8**  to  **UL5**  52 stimulated this activity in a dose dependent manner.  Ref: 8106478 J Biol Chem, 1994
- Transfection of combinations of subclones and of human cytomegalovirus immediate early promoter driven expression constructs for individual open reading frames led to the identification of six HSV genes which together were necessary and sufficient for the induction of DNA amplification UL30 DNA polymerase, UL29 major DNA binding protein,  **UL5** ,  **UL8** , UL42, and UL52.  Ref: 2547992 J Virol, 1989
- Moreover, these activities were also detected in cells in which only the  **UL5**  and UL52 products were expressed indicating that the presence of the  **UL8**  protein is essential for neither the ATPase nor helicase activity of the complex.  Ref: 2163521 Nucleic Acids Res, 1990
- Cotransfection of the cloned, replication competent AAV 2 genome together with the seven HSV replication genes  **UL5** ,  **UL8** , UL9, UL29, UL30, UL42, and UL52 led to productive AAV replication.  Ref: 1850024 J Virol, 1991
- The herpes simplex virus type 1  **UL5** ,  **UL8** , and UL52 genes encode an essential heterotrimeric DNA helicase primase that is responsible for concomitant DNA unwinding and primer synthesis at the viral DNA replication fork.  Ref: 9593724 J Biol Chem, 1998
- The herpes simplex virus HSV type 1 helicase primase is a three protein complex, consisting of a 1 1 1 association of  **UL5** ,  **UL8** , and UL52 gene products J.J.  Ref: 1321275 J Virol, 1992
- The assembly of complete HSV replication compartments and incorporation of BrdU were both abolished by treatment with phosphonoacetic acid and by omission of any one of the seven viral replication proteins,  **UL5** ,  **UL8** , UL9, UL42, UL52, SSB, and Pol, that are essential for viral DNA replication.  Ref: 9060678 J Virol, 1997
- The ability of the  **UL8**  mutants to facilitate efficient nuclear localization of UL52 in the presence of coexpressed  **UL5**  was examined by immunofluorescence.  Ref: 9344911 Virology, 1997
- To characterize the AAV functions mediating this effect, cloned AAV type 2 wild type or mutant genomes were transfected into simian virus 40 SV40 transformed hamster cells together with the six HSV replication genes encoding  **UL5** ,  **UL8** , major DNA binding protein, DNA polymerase, UL42, and UL52 which together are necessary and sufficient for the induction of SV40 DNA amplification R.  Ref: 2159559 J Virol, 1990
- Furthermore, since no detectable  **UL5**  protein is synthesized in hr99 infected cells, these cells provide a valuable control not only for the detection of the  **UL5**  protein itself but also for the detection of protein protein interactions with  **UL8**  and UL52 by coimmunoprecipitation.  Ref: 1309255 J Virol, 1992
- The ability of these three mutant  **UL5**  proteins to form specific interactions with other members of the helicase primase complex,  **UL8**  and UL52, indicates that the functional domains required for replication activity of  **UL5**  are separable from domains responsible for protein protein interactions.  Ref: 1309257 J Virol, 1992
- We also found that the primers made by the subcomplex are equal in length to those synthesized by the  **UL5**   **UL8**  UL52 complex.  Ref: 1321275 J Virol, 1992
- The UL6 locus of pseudorabies virus PRV was analyzed to reveal a gene cluster with homology to herpes simplex virus  **UL5** , UL6, UL7 and  **UL8** , Epstein Barr virus BBRF1 and BBRF2, and Kaposi sarcoma associated herpes virus ORF43 and ORF42.  Ref: 9458304 Oncol Rep< MedlineTA>Oncol Rep, 1992
- The Herpes simplex virus type 1 primosome consists of three subunits that are the products of the  **UL5** ,  **UL8** , and UL52 genes.  Ref: 9278436 J Biol Chem, 1997
- We observed that four replication proteins,  **UL5** ,  **UL8**  UL52, and UL9, are necessary for the localization of ICP8 UL29 to prereplicative sites natural infection conditions.  Ref: 8627698 J Virol, 1996
- On the basis of these results, we present a model for prereplicative site formation in infected cells in which the helicase primase components  **UL5** ,  **UL8** , and UL52, the origin binding protein UL9, and the viral single stranded DNA binding protein ICP8 assemble together to initiate the process.  Ref: 8627698 J Virol, 1996
- The recombinant enzyme which we have purified to near homogeneity from the insect cells has a molecular weight of 27 00 and is composed of the three polypeptides encoded by the  **UL5** ,  **UL8** , and UL52 genes.  Ref: 2556383 J Biol Chem, 1989
- The results demonstrated that all three components of the viral helicase primase complex  **UL5** ,  **UL8**  and UL52 proteins must be co expressed to allow their efficient localization to the nucleus.  Ref: 1312116 J Gen Virol, 1992
- The primase activity of the intertypic complex was 10 fold lower than that of HSV 1  **UL5**  HSV 1  **UL8**  HSV 1 UL52.  Ref: 9445019 J Virol, 1998
- Herpes simplex virus type 1 encodes a heterotrimeric helicase primase composed of the products of the  **UL5** , UL52, and  **UL8**  genes.  Ref: 9020191 J Biol Chem, 1997
- The herpes simplex virus helicase primase complex, a heterotrimer of the  **UL5** ,  **UL8** , and UL52 proteins, displays a single predominant site of primer synthesis on phi X174 virion DNA Tenney, D.  Ref: 7721827 J Biol Chem, 1995
- 26 0835 20838 we find that DNA dependent ATPase and DNA helicase activities are assembled in vivo in insect cells triply infected with viruses expressing the  **UL5** ,  **UL8**  and UL52 proteins.  Ref: 2163521 Nucleic Acids Res, 1990
- Herpes simplex virus type 1 HSV 1 encodes a heterotrimeric helicase primase composed of the products of the three DNA replication specific genes  **UL5** ,  **UL8** , and UL52 Crute, J.  Ref: 7775476 J Biol Chem, 1995
- We show that the HSV 1  **UL5** ,  **UL8** , UL29, UL30, UL42, and UL52 gene products along with the AAV Rep68 protein are sufficient to initiate replication on duplex DNA containing the AAV origins of replication, resulting in products several hundred nucleotides in length.  Ref: 11581393 J Virol, 2001
- Further characterization of the three subunit enzyme isolated from HSV 1 infected CV 1 cells shows it to be a heterotrimer, consisting of one polypeptide encoded by each of the  **UL5** ,  **UL8** , and UL52 genes.  Ref: 1847923 J Biol Chem, 1991
- Herpes simplex virus type 1 encodes a heterotrimeric helicase primase complex composed of the products of the  **UL5** , UL52, and  **UL8**  genes.  Ref: 11278618 J Biol Chem, 2001
- Herpes simplex virus type 1 HSV 1 encodes a helicase primase that consists of the products of the  **UL5** ,  **UL8** , and UL52 genes Crute, J.  Ref: 1847923 J Biol Chem, 1991
- The intertypic complex HSV 2  **UL5**  HSV 1  **UL8**  HSV 1 UL52 was purified and biochemically characterized.  Ref: 9445019 J Virol, 1998
- The herpes simplex virus type 1 DNA helicase primase is a heterotrimer encoded by the  **UL5** ,  **UL8** , and UL52 genes.  Ref: 8702953 J Biol Chem, 1996
- The  **UL5** ,  **UL8** , and UL52 genes of herpes simplex virus type 1 encode a multisubunit assembly that possesses primase, DNA helicase, and DNA dependent nucleoside triphosphatase activities.  Ref: 9013584 J Biol Chem, 1997
- These genes were designated  **UL5** ,  **UL8** , UL9, UL42, and UL52 and were predicted to encode proteins with molecular weights of, respectively, 9 0 0 0 00, and 11 00.  Ref: 2826807 J Virol, 1988
- The products of herpes simplex virus type 1 HSV 1 genes  **UL5** ,  **UL8**  and UL52 form a complex in virus infected cells that exhibits both DNA helicase and DNA primase activities.  Ref: 7931156 J Gen Virol, 1994
- The herpes simplex virus type 1 helicase primase complex consists of the products of the  **UL5** ,  **UL8**  and UL52 genes.  Ref: 2163521 Nucleic Acids Res, 1990
- Herpes simplex virus type 1 expresses a heterotrimeric helicase primase, the subunits of which are encoded by the viral  **UL5** ,  **UL8**  and UL52 genes.  Ref: 10501495 J Gen Virol, 1999
- While the  **UL8**  component of the helicase primase is not required for enzymatic activities by a subassembly of the  **UL5**  and UL52 proteins, only the holoenzyme  **UL5**  8 52 was stimulated by ICP8.  Ref: 9129659 J Gen Virol, 1997
- Since the  **UL5**  and UL52 proteins together form a complex which is enzymatically indistinguishable from a complex formed from all three proteins, a possible role of the  **UL8**  protein may be in facilitating nuclear uptake.  Ref: 1312116 J Gen Virol, 1992
- The results shown in this paper are consistent with the model that the five proteins, ICP8,  **UL5** ,  **UL8** , UL9, and UL52, form a scaffold.  Ref: 12634381 J Virol, 2003
- Immunochemical analysis has shown these polypeptides to be the products of three of the genes UL52,  **UL5** , and  **UL8**  that are required for replication of a plasmid containing a herpes simplex 1 origin oriS.  Ref: 2538835 Proc Natl Acad Sci U S A, 1989
- The ordered assembly of the herpes simplex virus HSV type 1 replication apparatus leading to replication compartments likely involves the initial assembly of five viral replication proteins, ICP8, UL9, and the heterotrimeric helicase primase complex  **UL5**   **UL8**  UL52, into replication foci.  Ref: 12634381 J Virol, 2003
- Cotransfections with different combinations of these genes demonstrated that a subset of four of them, coding for the HSV helicase primase complex  **UL5** ,  **UL8** , UL52 and the major DNA binding protein UL29, was already sufficient to mediate the helper effect.  Ref: 1850024 J Virol, 1991
- An assembly of the  **UL5**  and UL52 subunits retains both enzymic activities, and the  **UL8**  protein has been implicated in modulating these functions, facilitating efficient nuclear uptake of the complex and interacting with other viral DNA replication proteins.  Ref: 9344911 Virology, 1997
- The herpes simplex virus type 1 HSV 1 helicase primase, an essential component of the viral DNA replication machinery, is a trimeric complex of the virus coded  **UL5** ,  **UL8** , and UL52 proteins.  Ref: 9344911 Virology, 1997
- To further characterize the function of the  **UL8**  subunit, we have examined its effect on the activities of the  **UL5**  52 core enzyme using DNA templates covered by the herpes simplex virus type 1 single strand DNA binding protein ICP8.  Ref: 8702953 J Biol Chem, 1996
- Various immunological assays suggest that four of these proteins  **UL5** ,  **UL8** , UL9, and UL52 are made in infected cells in very low abundance relative to the other three.  Ref: 2535726 J Virol, 1989
- The purified  **UL8**  gene product, although required for viral DNA replication, neither exhibits these enzymatic activities nor stably associates with either the  **UL5**  or the UL52 gene product.  Ref: 1847509 Proc Natl Acad Sci U S A, 1991
- Herpes simplex virus 1 encodes a helicase primase that is composed of the products of the  **UL5** ,  **UL8** , and UL52 genes.  Ref: 1847509 Proc Natl Acad Sci U S A, 1991
- Herpes simplex virus type 1 encodes a heterotrimeric helicase primase complex that is composed of the products of the  **UL5** , UL52, and  **UL8**  genes.  Ref: 10075707 J Biol Chem, 1999
- Herpes simplex virus type 1 HSV 1 encodes a heterotrimeric helicase primase comprised of the products of the  **UL5** ,  **UL8** , and UL52 genes Crute, J.  Ref: 1657954 J Biol Chem, 1991
- the heterotrimeric helicase primase complex, encoded by  **UL5** ,  **UL8** , and UL52 genes, is believed to unwind duplex viral DNA at replication forks.  Ref: 11642367 Prog Nucleic Acid Res Mol Biol, 2001
